# Associating Intake Proportion of Carbohydrate, Fat, and Protein with All-Cause Mortality in Korean Adults

**DOI:** 10.3390/nu12103208

**Published:** 2020-10-21

**Authors:** Yu-Jin Kwon, Hye Sun Lee, Ju-Young Park, Ji-Won Lee

**Affiliations:** 1 Department of Family Medicine, Yongin Severance Hospital, Yonsei University College of Medicine, Gyeonggi 16995, Korea; digda3@yuhs.ac; 2 Biostatistics Collaboration Unit, Department of Research Affairs, Yonsei University College of Medicine, Seoul 03722, Korea; hslee1@yuhs.ac (H.S.L.); ka2pzy@naver.com (J.-Y.P.); 3 Department of Family Medicine, Gangnam Severance Hospital, Yonsei University College of Medicine, Seoul 06273, Korea

**Keywords:** carbohydrate, fat, protein, all-cause mortality

## Abstract

Determining the ideal ratio of macronutrients for increasing life expectancy remains a high priority in nutrition research. We aim to investigate the association between carbohydrate, fat, and protein intake and all-cause mortality in Koreans. This cohort study investigated 42,192 participants from the Korea National Health and Nutrition Examination Survey (KNHANES) linked with causes of death data (2007–2015). Hazard ratios (HRs) were calculated using the multivariable Cox proportional regression model after adjusting for confounders. We documented 2110 deaths during the follow-up period. Time to exceed 1% of the all-cause mortality rate was longest in participants with 50–60% carbohydrate, 30–40% fat, and 20–30% protein intake. Adjusted hazard ratio (HR) with 95% confidence intervals (CIs) was 1.313 (1.031–1.672, *p* = 0.0272) for <50% carbohydrate intake, 1.322 (1.116–1.567, *p* = 0.0013) for ≥60% carbohydrate intake, 1.439 (1.018–2.035, *p* = 0.0394) for <30% fat intake, and 3.255 (1.767–5.997, *p* = 0.0002) for ≥40% fat intake. There was no significant association between protein intake proportion and all-cause mortality. We found a U-shaped association between all-cause mortality and carbohydrate intake as well as fat intake, with minimal risk observed at 50–60% carbohydrate and 30–40% fat intake. Our findings suggest current Korean dietary guidelines should be revised to prolong life expectancy.

## 1. Introduction

Macronutrients (carbohydrate, fat, and protein) supply energy and influence various physiological functions [1]. Many studies have reported that specific dietary interventions can affect health-related outcomes and lifespan [2,3]. Increasing evidence has shown that calorie restriction without malnutrition increases lifespan by reducing the metabolic rate and improving insulin resistance, oxidative stress, and inflammation [4]. Calorie restriction retards the aging processes through hormetic action by acting as low-intensity stress [5]. Hormesis in aging refers to beneficial effects resulting from the cellular responses to mild and repeated stress [6]. Recent works have suggested that macronutrient composition and balance, rather than total energy intake, contribute to expanded lifespan [7,8,9].

Nutritional dietary guidelines recommend the ideal energy and macronutrient intake to improve public health and prevent chronic diseases. The Institute of Medicine has developed a new approach for establishing recommended dietary allowances for macronutrients [10,11]. The 2015–2020 Dietary Guideline for Americans recommends that carbohydrates comprise 45–65% of calories, fat 25–35% of calories, and protein 10–30% of calories [12].

Data associating macronutrient intake proportion and all-cause mortality remain conflicting [13,14]. The Prospective Urban Rural Epidemiology (PURE) study reported that high carbohydrate intake was associated with higher total mortality risk, whereas total fat intake was related to lower total mortality risk [13]. The Atherosclerosis Risk in Communities (ARIC) study showed a U-shaped association between carbohydrate intake and mortality [14]. A large prospective cohort study conducted in the US reported higher mortality with high saturated fatty acids, trans-fatty acids, animal monounsaturated fatty acids, α-linolenic acid, and arachidonic acid intake [15].

Dietary habits differ across countries, races, and cultures. Asians experienced rapid economic and nutrition transition [16]. In South Korea, carbohydrate intake decreased and fat intake increased, while protein intake remained constant during rapid economic growth [16]. Changes in eating patterns affect the occurrences of lifestyle-related diseases, such as obesity, metabolic syndrome, and cardiovascular diseases [17]. However, studies assessing macronutrient intake and all-cause mortality in Asians remain lacking.

Therefore, we investigated the association between carbohydrate, fat, and protein intake with all-cause mortality using a large representative Korean dataset. Results from the study were aimed to propose an appropriate macronutrient intake ratio to reduce all-cause mortality.

## 2. Materials and Methods

### 2.1. Study Population

**Figure 1:**
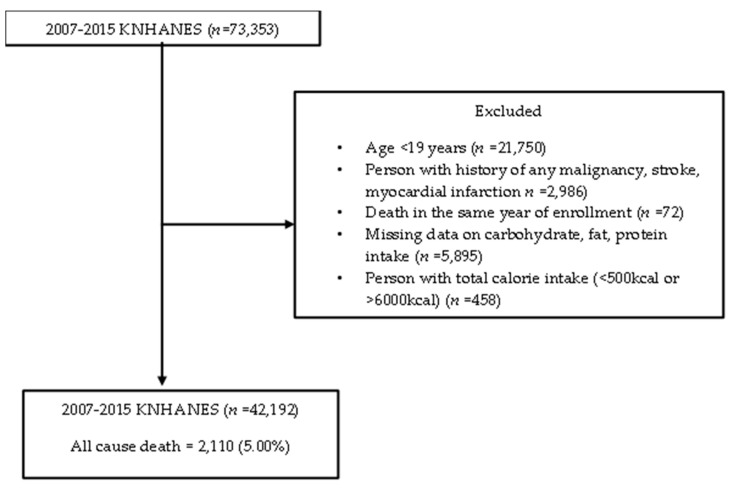
Flow chart of the study population.

Population data came from the Korea National Health and Nutrition Examination Survey (KNHANES) linked with causes of death data (version 1.1) from Statistics Korea. KNHANES is an ongoing surveillance system in the Republic of Korea that assesses the health and nutritional status of Koreans, monitors trends in health risk factors, and provides data for the development and evaluation of health policies in Korea. Detailed information on KNHANES is available at: https://knhanes.cdc.go.kr/knhanes/eng. Permission was obtained in March 2020 from the Korea Centers for Disease Control and Prevention for the data linking the causes of death from Statistics Korea with the KHANES data. Only participants from the 2007–2015 study period aged 19 or older who agreed to link their statistics with the causes of death from Statistics Korea were included in this study. A total of 73,353 participants were enrolled in the 2007–2015 KNHANES. We excluded participants under 19 years of age (*n* = 21,750); with a history of any malignancy, stroke, or myocardial infarction (*n* = 2986); death in the same year of enrollment (*n* = 72); missing carbohydrate, fat, protein intake data (*n* = 5895); and with total calorie intake <500 kcal or >6000 kcal (*n* = 458). A total of 42,192 participants were included in the final analysis. Figure 1 presents a flow chart of the study population. Informed consents were obtained before participating in the KNHANES. This study was approved by Gangnam Severance Hospital Institutional Review Board (no. 3-2020-0093).

### 2.2. Assessment of Nutritional Status

The nutrition survey was conducted with face-to-face interviews by trained medical staff. The food intake questionnaire was designed as an open-ended survey for reporting various dishes and foods using the 24-hour recall method with various measuring aids. The survey staff were required to complete an intensive training course and supervised practice before working in the survey field. Total grams of carbohydrate, fat, protein, and alcohol were calculated from the 24-hour dietary recall. Total consumption of carbohydrates, protein, and fat was then converted to energy intake in calories (1 g carbohydrates = 4 kcal, 1 g protein = 4 kcal and 1 g fat = 9 kcal). The proportion of carbohydrates, protein, and fat intake was calculated as follows: carbohydrate, protein, and fat intake calories/total calorie intake × 100.

### 2.3. Ascertainment of All-Cause Mortality

Mortality status and cause of death were determined using the KNHANES-linked National Death Index public-access files. Participant deaths were tracked from initial assessment to 2017. Underlying causes of death were defined in the Korean Standard Classification of Diseases (KCD) code based on the National Death Index. All-cause mortality included all specified and unknown causes.

### 2.4. Covariates

According to standardized protocols, all health examination procedures were performed by trained medical personnel and all equipment calibrated periodically. Height was obtained to the nearest 0.1 cm with participants wearing light indoor clothing and weight to the nearest 0.1 kg with no shoes. Body mass index was calculated as: weight in kilograms/height in meters squared (kg/m^2^). Blood pressures were measured twice in a sitting position. Blood tests were conducted after a minimum of eight hours of fasting. Smoking, alcohol intake, and physical activity were determined by self-reported questionnaire. Smokers were defined as those who reported smoking more than 100 cigarettes in a lifetime and were still actively smoking. Alcohol drinkers were defined as those who still actively drank alcohol. Physical activity was assessed using the Korean version of the Global Physical Activity Questionnaire, and regular exercisers were defined as individuals who walked at least 0.5 h per day for more than five days per week.

### 2.5. Statistical Analysis

We had analyzed the data based on the law of large numbers and central limit theorem. Differences in general characteristics of all-cause mortality events were presented as mean and standard deviation (SD) or number (%). Results were compared in an independent two sample *t*-test for continuous variables and chi-squared test for categorical variables. A density plot of carbohydrate, fat, and protein intake was drawn at 10 percent increments with the mean ± SD and median (interquartile range) for each macronutrient summarized. A Cox proportional hazard spline plot was used to assess associations between macronutrients (carbohydrate, fat, and protein) and all-cause mortality events. The reference range was set based on the lowest point of the curve: 50–60% for carbohydrate, 30–40% for fat, and 20–30% for protein intake. Warranty period was defined as the time to reach the cumulative mortality rate >1% for each group. If the 1% threshold was not met, the value was expressed as the time of the last follow-up. Incidence per 1000 person-years was calculated for each group. Hazard ratios (HRs) were calculated using the multivariable Cox proportional regression model. All multivariable models were adjusted for age, sex, BMI, total calorie, smoking, alcohol, and exercise. A forest plot indicated the hazard ratio and 95% confidence intervals. All statistical analyses were performed using SAS software, version 9.4 (SAS Institute Inc., Cary, NC, USA), and R software, version 3.5.2 (The R Foundation for Statistical Computing, Vienna, Austria; http://www.R-project.org/). All statistical tests were two-sided and statistical significance set at *p* = 0.05.

## 3. Results

Table 1 shows the baseline characteristics of the study population at follow-up. A total of 42,192 participants were included in this study. We documented 2110 deaths with mean age ± SD of 70.2 ± 11.1 years during the follow-up period (labelled as “event group”). The remaining “non-event” participants averaged 48.7 ± 15.9 years. The event group had significantly more men than the non-event group. Mean BMI ± SD was 23.0 ± 3.4 in the event group and 23.7 ± 3.4 in the non-event group. The mean systolic blood pressure, pulse rate, fasting glucose, and triglyceride were significantly higher in the event group than the non-event group. The mean diastolic blood pressure, total cholesterol, and HDL-cholesterol were significantly lower in the event group than the non-event group. The event group had significantly more smokers but significantly fewer alcohol drinkers than the non-event group. The number of regular exercisers did not differ between the two groups.

Nutritionally, total energy intake was 1630.8 ± 675.1 kcal/day for the event group and 1951.2 ± 813.8 kcal/day for the non-event group (*p* < 0.0001). The proportion of carbohydrate intake (73.0 ± 12.6% vs. 66.5 ± 13.3%, *p* < 0.0001) was significantly higher but the proportions of fat intake (11.8 ± 7.4% vs.17.2 ± 8.7%, *p* < 0.0001) and protein intake (12.7 ± 3.9% vs. 14.0 ± 4.0%, *p* < 0.0001) significantly lower for the event group versus the non-event group.

**Figure 2:**
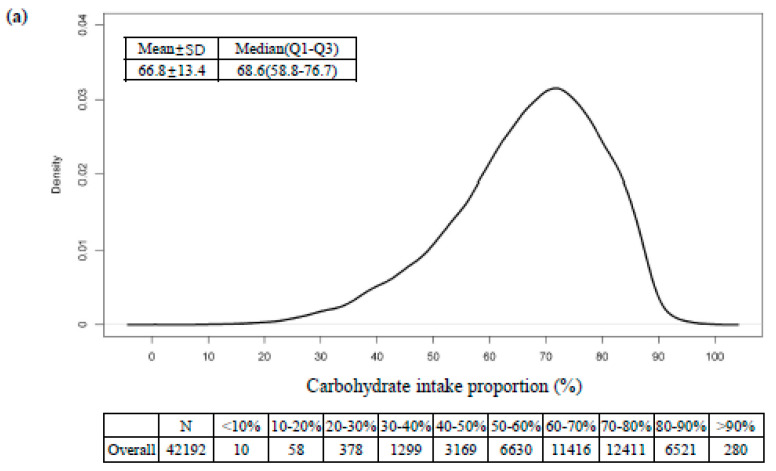

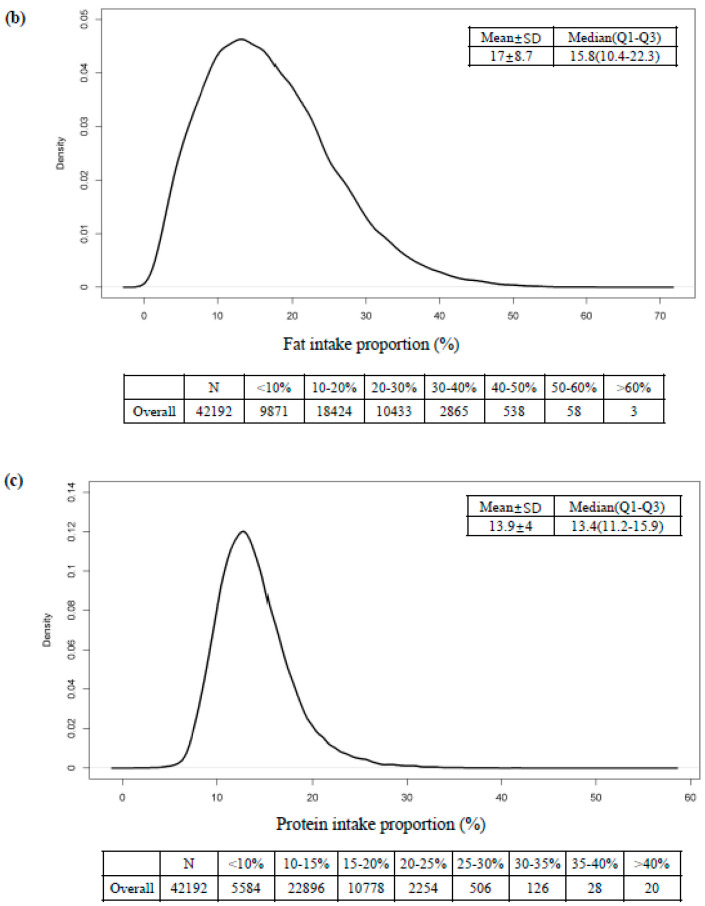
Density plot of estimated (**a**) carbohydrate, (**b**) fat, and (**c**) protein intake proportion in Korean adults.

Figure 2 presents the distribution of dietary carbohydrate, fat, and protein intake. The mean ± SD and median (IQR) levels were 66.8 ± 13.4 and 68.6 (58.8, 76.7) for carbohydrate (%), 17.0 ± 8.7 and 15.8 (10.4, 22.3) for fat (%), and 13.9 ± 4.0 and 13.4 (11.2, 15.9) for protein (%).

**Figure 3:**
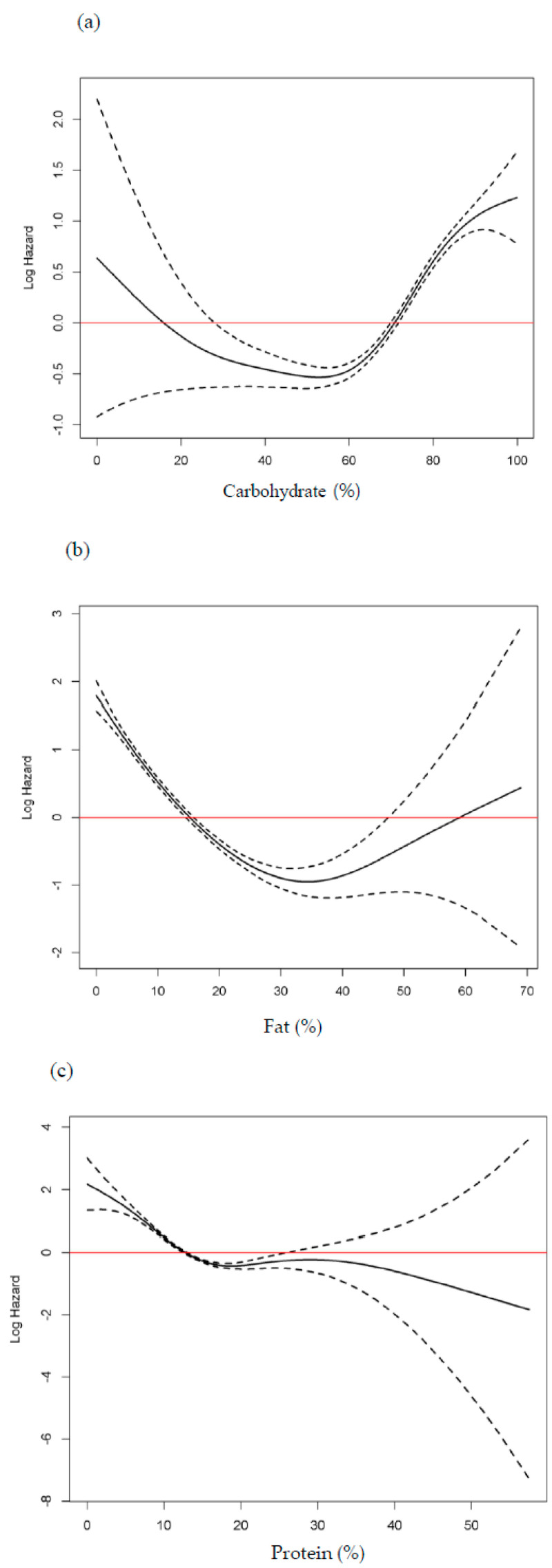
Cox proportional hazard spline curve of (**a**) carbohydrate, (**b**) fat, and (**c**) protein intake proportion for total mortality. Solid lines represent the nonlinear spline models and dashed lines indicate their confidence intervals. Red line indicate that hazard ratio is one.

We used the Cox proportional hazard spline curve to associate carbohydrate, fat, and protein intake proportion with all-cause mortality (Figure 3). All three macronutrients exhibited U-shaped associations with all-cause mortality. The nadir points of all-cause mortality were at approximately 40–60% for carbohydrate intake and 30–40% for fat intake. All-cause mortality increased when protein intake was less than 10%.

Table 2 shows the warranty periods and incidences per 1000 person-years (95% CIs) for all-cause mortality with reference to carbohydrate, fat, and protein intake proportion. The proportions with the longest warranty period for exceeding 1% of the all-cause mortality rate was 50–60% carbohydrate for 3.5 years, 30–40% fat for 6.2 years, and 20–30% protein for 2.8 years.

**Figure 4:**
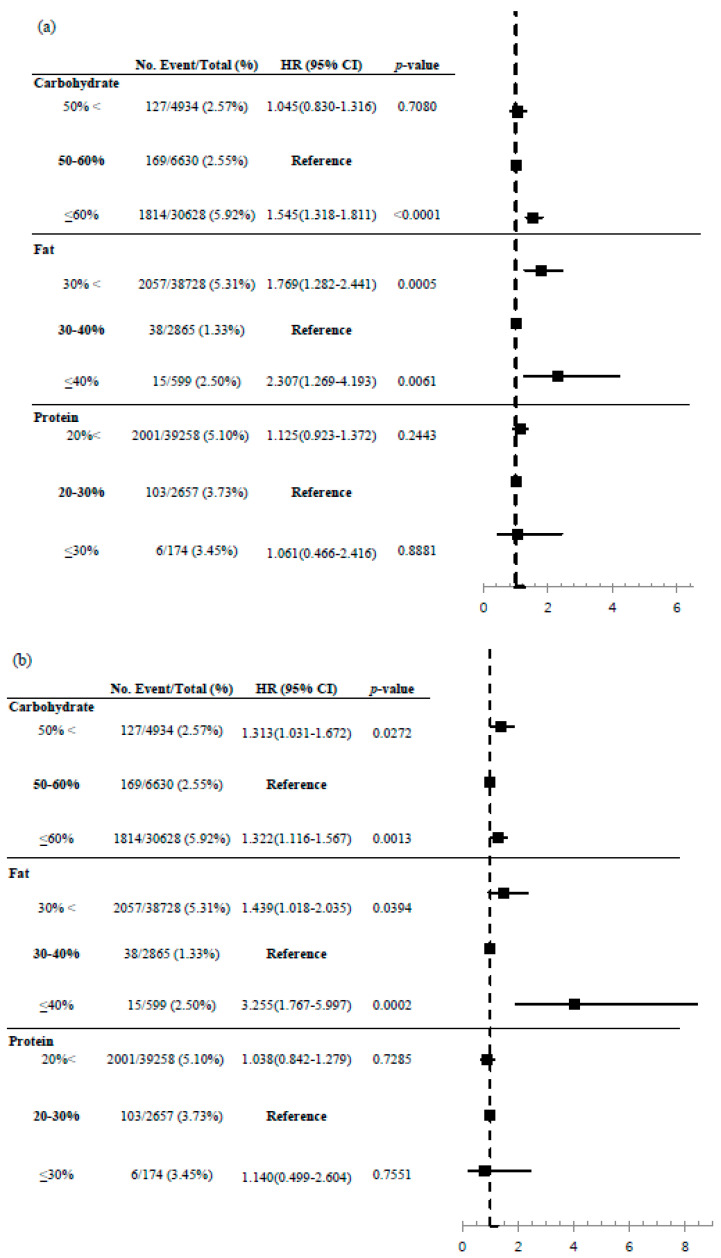
Forest plot of carbohydrate, fat, and protein proportion for total mortality. (**a**) adjusted hazard ratio (HR) and 95% confidence intervals (CI) after adjusting for age and sex (**b**) adjusted hazard ratio (HR) and 95% confidence intervals (CI) after adjusting for age, sex, body mass index, total calorie, smoking, alcohol, and exercise.

Figure 4 uses the intake ranges with the longest warranty periods to draw a forest plot. When 50–60% of carbohydrate intake was taken as reference, the HR and 95% CIs, after adjusting for age and sex, was 1.045 (0.830–1.316, *p* = 0.078) for less than 50% carbohydrate intake and 1.545 (1.318–1.811, *p* < 0.001) for over 60% carbohydrate intake. When age, sex, BMI, total calorie, smoking, alcohol drinking, and exercise were adjusted, the HR and 95% CIs became 1.313 (1.031–1.672, *p* = 0.0272) for less than 50% carbohydrate intake and 1.322 (1.116–1.567, *p* = 0.0013) for over 60% carbohydrate intake.

When 30–40% of fat intake was taken as reference, the HR and 95% CIs, after adjusting for age and sex, was 1.769 (1.282–2.441, *p* = 0.0005) for less than 30% fat intake and 2.307 (1.269–4.193, *p* = 0.0061) for over 40% fat intake. When age, sex, BMI, total calorie, smoking, alcohol drinking, and exercise were adjusted, the HR and 95% CIs became 1.439 (1.018–2.035, *p* = 0.0394) for less than 30% and 3.255 (1.767–5.997, *p* = 0.0002) for over 60% fat intake.

With protein intake, no significant association was found between protein intake and all-cause mortality with 20–30% intake as the reference.

## 4. Discussion

Nutrition has a substantial impact on health and life expectancy, and the effect of macronutrient intake on health still interests physicians and public health professionals. Although many studies have tried to clarify the effect of carbohydrate, fat, and protein intake on all-cause mortality, controversies remain [14,18,19,20].

A recent prospective cohort study using both population-based and pooled previous prospective studies reported that a low carbohydrate diet was significantly associated with overall and cause-specific mortality [21]. However, the PURE study in 2017 involving 18 countries across five continents found contradictory results with higher carbohydrate intake associated with higher mortality risk [13]. The ARIC study conducted in 2018 of four US communities observed a U-shaped association between carbohydrate intake and total mortality, with 50–55% carbohydrate intake yielding the longest lifespan [14]. This result was in line with our study. We found a nonlinear association between carbohydrate intake and all-cause mortality. Compared to participants with 50–60% carbohydrate intake, a diet with <50% or >60% energy from carbohydrate and <30% or >40% energy from fat was associated with significant all-cause mortality risk. Although 20–30% energy intake from protein yielded the longest warranty period, there was no significant association between protein intake proportion and all-cause mortality. In 2015, the Korean Nutrition Society lowered the acceptable macronutrient distribution range (AMDR) of carbohydrates from 55–70% in 2010 to 55–65%, elevated the AMDR of fat from 15–25% to 15–30%, and maintained the AMDR of protein at 7–20% in adults over 19 years of age [22]. However, the Korean population still consumed a higher proportion of carbohydrate and lower proportion of fat than the Western population [23]. Therefore, our findings suggest lowering the current recommended Korean AMDR of carbohydrate by five percent.

The low carbohydrate, high fat diet has become popular in recent years for various health outcomes ranging from weight reduction to longevity [24]. Carbohydrate restricted diets applied in clinical practice include: Akins (restricting only carbohydrate, but not protein and fat), South Beach and Zone (moderate carbohydrate with moderate protein and fat), and Ketogenic (very low carbohydrate and restricted protein and fat) [2]. The long-term safety of consuming a high-fat diet remains controversial. The PURE study reported that higher total fat intake was associated with lower mortality [13]. The PREvención con DIeta MEDiterránea (PREDIMED) study, which included participants with cardiovascular diseases (CVD) risk, also reported higher total fat intake with lower risk of mortality [25]. A considerable proportion of the total fat consumed in the study population living in the Mediterranean area originated from healthy fats (monounsaturated and polyunsaturated fatty acids) [26]. The well-known Mediterranean diet touted for preventing non-communicable diseases consists of at least 40% intake from fats, originating mainly vegetables and fish [26,27]. A large prospective cohort data from the National Institutes of Health-AARP Diet and Health Study found that saturated fat, monounsaturated fat from animal sources, and trans-fatty acid intake were associated with higher mortality, whereas plant monounsaturated fats and marine omega-3 polyunsaturated fats were associated with lower mortality [15]. Surprisingly, our study found 30–40% fat intake was the nadir point for total mortality. Both low (<30%) and high (≥40%) fat intake was associated with increased total mortality. Our results suggest that elevating healthy fat intake consumption within an optimal range could help increase life expectancy.

Study results associating protein intake with mortality are inconsistent. Levin et al. [28] showed that consuming large amounts of animal protein increases Insulin growth factor-1 (IGF-1) and insulin, major promoters of aging and mortality. A meta-analysis of in vitro studies concluded that restricting protein, rather than calories, prolong life expectancy [29]. However, several epidemiological studies noted that different types of protein (animal or plant-based) yield different health outcomes [20,30]. Two recent meta-analyses reported that higher total protein intake from mainly animal protein increased all-cause mortality, whereas plant protein decreased all-cause mortality [20,30]. The different effects between animal and plant proteins might be attributed to differing amino acid composition. Animal proteins contain more branched-chain and aromatic amino acids that are related to increased serum IGF-1 and insulin [7]. Plant protein have relatively lower essential amino acids, specifically leucine, lysine, and methionine [31].

Our study found insignificant association between total protein and all-cause mortality. This might be from our inability to distinguish the protein source. Another possible explanation is the different dietary habits between Koreans and Westerners. Traditional Korean foods are characterized by high carbohydrates, low animal protein and fat, and plenty of plant foods [32]. Older Koreans are particularly known to consume more than two-thirds of protein intake from plants (grains, legumes, and vegetables) [33].

Our study has several limitations. First, 24-hour diet recalls were used and might not fully reflect participants’ typical diet. To reduce this bias, a random nutrition survey schedule was used to include weekdays and weekends. Second, we only assessed carbohydrate, protein, and fat intake without considering food source type. For example, we could not distinguish between whole and refined grain, saturated and unsaturated fat, or animal and plant protein. However, we designed this study to suggest the optimal macronutrient ratio for the general population. Lastly, our results cannot be generalized to other ethnic groups with different dietary habits.

Despite these limitations, our study has strengths. We are the first to investigate the optimal macronutrient ratio using a large population-based prospective Korean cohort data. Research studies typically use quartiles or quintiles of macronutrients, but our study used proportions after verifying continuous exposure with spline plots. We further identified the relative risk of each proportion by calculating the warranty period to 1% of the all-cause mortality rate.

## 5. Conclusions

This study found that 50–60% energy intake from carbohydrates and 30–40% from fat were associated with decreased all-cause mortality risk in the Korean population. Although protein intake was not associated with all-cause mortality in Korean, the longest warranty period to 1% all-cause mortality risk was with 20–30% energy intake from proteins. Our findings suggest that well-balanced macronutrient consumption can help prolong lifespan. Furthermore, revising the current Korean dietary guideline for macronutrients ratio should be considered.

## Figures and Tables

**Table 1:** Baseline characteristics of study population at follow-up (2007–2015 KNHANES).

	All-Cause Mortality		
Variables	Event (*n* = 2110)	Non-Event (*n* = 40,082)	*p*-Value
Age	70.2 ± 11.1	48.7 ± 15.9	<0.0001
Sex			<0.0001
Female	936 (44.4)	24,309 (60.7)	
Male	1174 (55.6)	15,773 (39.3)	
BMI, kg/m^2^	23.0 ± 3.4	23.7 ± 3.4	<0.0001
Smokers			<0.0001
No	871 (45.6)	25,271 (65.0)	
Yes	1041 (54.4)	13,585 (35.0)	
Alcohol drinkers			<0.0001
No	1212 (73.5)	19,691 (54.2)	
Yes	436 (26.5)	16,643 (45.8)	
Exercise			0.0566
No	1326 (62.8)	24,356 (60.8)	
Yes	784 (37.2)	15,726 (39.2)	
SBP, mmHg	129.2 ± 19.4	118.1 ± 17.2	<0.0001
DBP, mmHg	74.7 ± 11.5	75.6 ± 10.5	0.0006
Pulse rate	66.7 ± 20.2	58.4 ± 13.2	<0.0001
Fasting Glucose, mg/dL	107.4 ± 35.4	97.8 ± 22.0	<0.0001
Total cholesterol, mg/dL	187.0 ± 39.3	189.0 ± 35.7	0.0363
Triglycerides, mg/dL	140.1 ± 109.8	131.6 ± 104.4	0.0016
HDL-cholesterol, mg/dL	46.2 ± 12.3	49.9 ± 11.8	<0.0001
Total Energy intake	1630.8 ± 675.1	1951.2 ± 813.8	<0.0001
Carbohydrate (%)	73.0 ± 12.6	66.5 ± 13.3	<0.0001
Fat (%)	11.8 ± 7.4	17.2 ± 8.7	<0.0001
Protein (%)	12.7 ± 3.9	14.0 ± 4.0	<0.0001

KNHANES, Korea National Health and Nutrition Examination Survey; BMI, Body mass index, SBP, Systolic blood pressure; DBP, Diastolic blood pressure; HDL, High density lipoprotein. Data are presented as mean ± standard deviations or number (%). *p*-values were calculated using the independent *t*-test or Chi-squared test. Carbohydrate (%) = carbohydrate (g) × 4 kcal/total energy intake (kcal) × 100, Fat (%) = fat (g) × 9 kcal/total energy intake (kcal) × 100, Protein (%) = protein (g) × 4kcal/total energy intake (kcal) × 100.

**Table 2:** Warranty periods for all-cause mortality according to carbohydrate (%), fat (%), and protein (%).

Variables	Warranty Period (1%)	*n*	Person-Time (Years)	Events, *n* (%)	Incidence Per 1000 Person-Years (95% CI)
Carbohydrate (%)					
<40%	3.422	1765	12,113.74	50(2.8)	4.128(2.986–5.269)
40–50%	3.499	3169	21,895.57	77(2.4)	3.517(2.733–4.301)
50–60%	3.504	6630	46,056.25	169(2.6)	3.669(3.117–4.222)
60–70%	3.170	11,416	82,012.67	360(3.2)	4.390(3.937–4.842)
≤70%	1.918	19,212	141,478.84	1454(7.6)	10.277(9.752–10.803)
Fat (%)					
<10%	1.584	9871	71,833.02	1029(10.4)	14.325(13.456–15.194)
10–20%	2.748	18,424	134,497.21	815(4.4)	6.060(5.645–6.474)
20–30%	4.255	10,433	73,652.93	213(2.0)	2.892(2.504–3.280)
30–40%	6.167	2865	19,461.84	38(1.3)	1.953(1.332–2.573)
≤40%	2.666	599	4112.05	15(2.5)	3.648(1.805–5.490)
Protein (%)					
<10%	1.586	5584	38,546.72	531(9.5)	13.775(12.612–14.939)
10–20%	2.674	33,674	243,031.97	1470(4.4)	6.049(5.740–6.357)
20–30%	2.836	2760	20,725.99	103(3.7)	4.970(4.012–5.927)
≤30%	1.923	174	1252.38	6(3.5)	4.791(0.967–8.615)

CI, Confidence interval.
